# Passive sampling devices enable capacity building and characterization of bioavailable pesticide along the Niger, Senegal and Bani Rivers of Africa

**DOI:** 10.1098/rstb.2013.0110

**Published:** 2014-04-05

**Authors:** Kim A. Anderson, Dogo Seck, Kevin A. Hobbie, Anna Ndiaye Traore, Melissa A. McCartney, Adama Ndaye, Norman D. Forsberg, Theodore A. Haigh, Gregory J. Sower

**Affiliations:** 1 Environmental and Molecular Toxicology Department, Oregon State University, ALS 1007, Corvallis, OR 97331, USA; 2 Centre Régional de Recherches en Ecotoxicologie et de Sécurité Environnementale (CERES), Km 15, Route de Rufisque, Dakar BP 3300, Sénégal

**Keywords:** polyethylene samplers, mixtures, polycyclic aromatic hydrocarbons, sub-Saharan Africa, food security, contaminant screening

## Abstract

It is difficult to assess pollution in remote areas of less-developed regions owing to the limited availability of energy, equipment, technology, trained personnel and other key resources. Passive sampling devices (PSDs) are technologically simple analytical tools that sequester and concentrate bioavailable organic contaminants from the environment. Scientists from Oregon State University and the Centre Régional de Recherches en Ecotoxicologie et de Sécurité Environnementale (CERES) in Senegal developed a partnership to build capacity at CERES and to develop a pesticide-monitoring project using PSDs. This engagement resulted in the development of a dynamic training process applicable to capacity-building programmes. The project culminated in a field and laboratory study where paired PSD samples were simultaneously analysed in African and US laboratories with quality control evaluation and traceability. The joint study included sampling from 63 sites across six western African countries, generating a 9000 data point pesticide database with virtual access to all study participants.

## 1. Introduction

Characterization of pesticides and other contaminants in aquatic systems in remote regions of less-developed countries presents many challenges. Lack of infrastructure, electricity, technology, trained personnel or controlled sample transportation or storage, in addition to harsh climatic conditions can make traditional environmental sampling impractical. Remote sites may require extended transportation times back to the laboratory coupled with ambient temperatures that are not amenable to preserving water samples. In addition to these situational challenges, pesticides are often applied episodically meaning that pesticide residues may go undetected in river systems if samples are only taken at one time point as with grab-sampling. Generally, composite sampling is too laborious and cost-intensive to be feasible. The challenges associated with sampling and a lack of adequate, accessible laboratories and/or pesticide analysis technical expertise have contributed to the scarcity of current data on pesticides and other contaminants of concern in the African agricultural systems of the Niger and Senegal Rivers [1–3].

Conventional methods of assessing human and ecological exposure and risk involve measuring contaminant concentrations in the ambient environment and extrapolating to toxicological endpoints; however, this approach has proved ineffective [4,5]. Contaminant concentrations measured directly in water often do not correlate well with tissue concentrations in species of concern [6]. Employing passive sampling devices (PSDs) addresses some of these concerns. Passives sampler membranes mimic contaminant uptake by a cell or organism through selective chemical and physical processes [7]. PSDs can be used to assess exposure and contamination in water [8,9]. The freely dissolved (*C*_free_) forms of hydrophobic contaminants can be absorbed by or moved through biological membranes of organisms, where they may exert toxic effects [10,11]. PSDs mimic these passive uptake processes; they sequester and concentrate contaminants, providing time-integrated measures of bioavailable (*C*_free_) contaminant load [12].

Passive sampling has a number of additional advantages over other sampling methods. Because PSDs continuously accumulate chemicals while deployed in the environment, concentrations of contaminants in PSDs are orders of magnitude greater than environmental concentrations. This greatly reduces the sensitivity needed for the analytical detection of low concentration contaminants, facilitating instrumental analysis in less-advanced laboratories and by novice analysts. Because PSDs sequester chemicals during the entire time that they are deployed, deployments can last from hours to days, weeks or months depending on the study needs. This allows PSDs to capture less frequent, acute, episodic contamination events that could be missed during periodic grab-sampling. The devices weigh only a few grams and are therefore easy to transport. Additionally, many contaminants of concern do not degrade in PSDs at ambient conditions allowing for unrefrigerated transportation [13]. Overall, PSDs are ideally suited for determining the bioavailability of chemicals, quantifying chemicals that are present at low concentrations in water and capturing episodic events [7,14,15]. They are uniquely appropriate for environmental monitoring in remote or less-developed areas.

Sub-Saharan West Africa requires improved food security driven by a sustainable increase in food production [16,17]. Most countries along the Senegal and Niger Rivers will likely maximize the irrigable lands for rice and vegetables. This could result in increased chemical use. This increase will come from expanding agriculture into new areas and increasing productivity in existing farms. Both approaches will need to be undertaken in concert with the improved capacity to adaptively manage agriculture at multiple scales, from the individual crop to complete production systems. Adaptive approaches require that feedback from agricultural production is available to farmers, extension workers, regulators and policymakers. The most important feedback is from reliable data that elucidate the current status and trends of agricultural production. One element of this feedback is relevant spatially and temporally resolved pesticide-monitoring information. Further development of regional laboratories that can generate these kinds of data is therefore an equally important priority.

Passive samplers were employed as part of training, capacity building and technology exchange between Oregon State University's (OSU) Food Safety and Environmental Stewardship Laboratory and the environmental chemistry laboratory of the Centre Régional de Recherches en Ecotoxicologie et de Sécurité Environnementale (CERES). OSU researchers and technicians trained colleagues from CERES in the construction of PSDs, their proper deployment in the field, and their subsequent extraction and analysis in accordance with a quality assurance (QA) plan. The training culminated in two sampling campaigns where OSU and CERES deployed paired PSDs at a total of 63 field sites during winter and autumn of 2011. CERES analysed one set of samples and OSU analysed the other set, providing quality control (QC) and training assessment when the results were compared. This work generated over 9000 pesticide concentration data points between the two laboratories. To the authors' knowledge, this is the first published field and laboratory study where duplicate field samples were collected and simultaneously analysed in African and US laboratories in support of a human and ecological risk study, with QC evaluation and traceability.

In addition to detailing the training and scientific capacity-building elements of the project, this paper examines the occurrence of pesticides and other contaminants of concern at the study sites. Samples from engineered, irrigated agricultural systems in Mali, Mauritania, Guinea, Niger, Benin and Senegal were analysed for bioavailable pesticides. Samples were also screened for over 1100 contaminants of concern using mass spectral de-convolution reporting software (DRS). The screening method included polycyclic aromatic hydrocarbons (PAHs), substituted PAHs (methyl-, oxy- and nitro-PAHs), pesticides, polychlorinated biphenyls (PCBs), personal care products and contaminant metabolites.

This project had two major elements, and thus the objectives are twofold. The objectives related to building scientific and technical capacity in African laboratories are: (i) to provide the foundation necessary to properly conduct integrated environmental monitoring, chemical analysis and human and ecological health risk assessment; (ii) to contribute to building and strengthening in-country capacities to enable local, national and regional environmental monitoring that would generate the high-quality data necessary for setting protective standards for human and environmental health; and (iii) to contribute to the development of a robust model for sustainable agricultural production, including ecological modelling and human health modelling/assessments, as discussed further in Jepson *et al.* [18] and Settle *et al.* [19]. The objectives of the environmental assessment component of this project are: (i) to demonstrate the utility of PSDs for large-scale assessment of bioavailable contaminants and (ii) to characterize the contaminants in agricultural systems of two major West African rivers to support future human and ecological health risk assessment.

## 2. Material and methods

### (a) Capacity building

**Figure 1.**
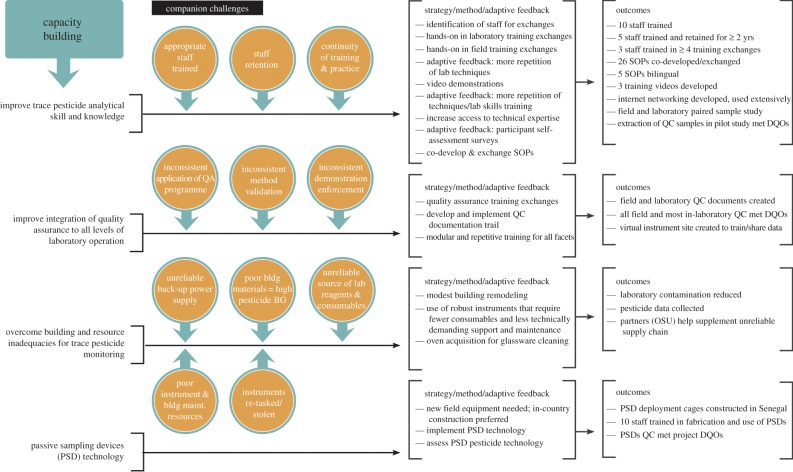
Capacity-building needs, companion challenges, strategies, methods and outcomes: summarizes the needs assessment, recognizes previous and associated challenges, the approach and adaptive feedback, and the final outcomes. bldg, building; maint., maintenance; BG, background; DQOs, data quality objectives.

Through a participatory process between CERES and OSU, capacity-building needs were identified (figure 1). The needs assessment focused on three components: trace pesticide analytical skills and knowledge, integration of QA and passive sampling. After initial assessments, building and chemical resource inadequacies were added as capacity-building needs. As highlighted in figure 1, other acknowledged challenges were considered in the development of the capacity-building process. The methods used to address capacity building are also summarized in figure 1. Capacity building included foundational concepts but focused on hands-on practical training. Each hands-on training module included some components of the three major training themes: QA, passive sampling and pesticide analysis; see the electronic supplementary material, table S1 for specific training events and focus topics. Each training event included PSD fabrication and practice fieldwork, and all training activities integrated opportunities to teach QA/QC processes. Additionally, several learning tools were developed and made available to researchers at CERES to help to facilitate knowledge transfer, including hard-copy training manuals that included QA procedures, an online web portal on a public OSU website (http://fses.oregonstate.edu), training videos that provided instruction on PSD cleaning and extraction and standard operating procedures (SOPs) that detailed analysis. When training onsite, researchers used web logs, particularly Google Blogger, to document training and conditions. Adaptive feedback and fine-tuning of the process were implemented as the project demanded.

As part of the adaptive feedback for training, OSU prepared a self-assessment survey for CERES staff to complete before and after training (see electronic supplementary material). The survey aimed to determine the strengths and weaknesses of the programme through participant self-assessment. The survey consisted of 20 questions provided in both English and French. Trainees were asked to answer each question based on a 1–7 scale, where 1 indicated no ability or skill and 7 indicated very high degree of skill or ability. Survey questions pertained to QA, fabrication and use of PSDs, field sampling, quantitative analytical skills, analytical instrumental analysis and data interpretation. Training coordinators also evaluated each event.

In addition to self-surveys, quantitative analytical results for paired samples were directly compared to evaluate the efficacy of training. Analytical and technical capacity was assessed using an enhanced data management strategy hosted by OSU; details are provided in the electronic supplementary material.

### (b) Passive sampling device fabrication

PSDs were constructed from low-density polyethylene tubing using methods detailed elsewhere [20]. To determine *in situ* uptake rates [7,13,21–23], PSDs were fortified with PCB-100, PCB-180 and pentachloronitrobenzene as performance reference compounds (PRCs) prior to sealing the tubing. PSD fortification took place at both OSU and CERES.

### (c) Sample collection

#### (i) Study area

**Figure 2.**
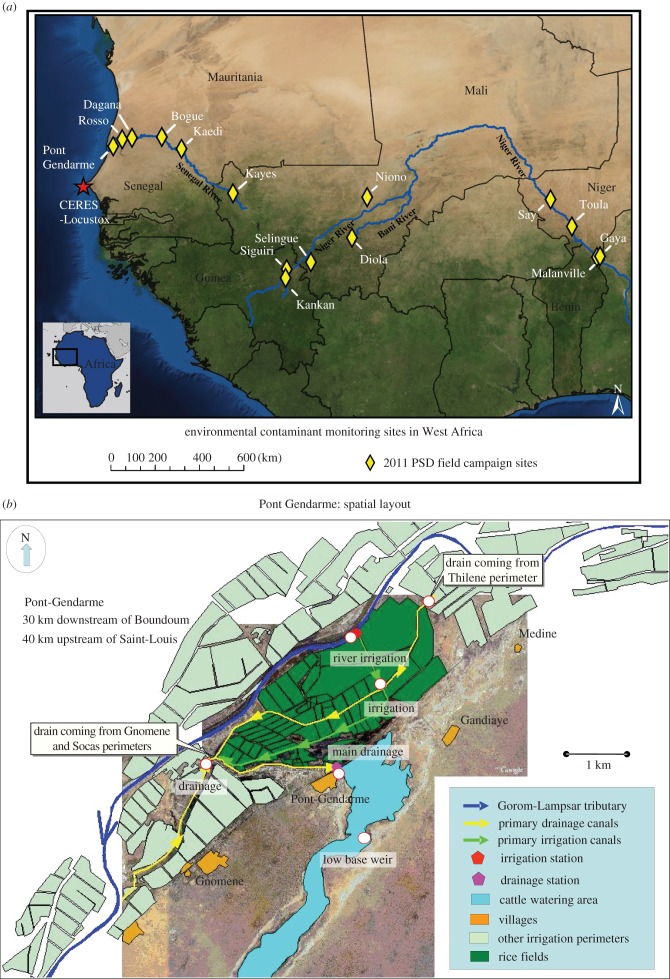
Study area and sampling sites: (*a*) all field campaign sites within the Senegal, Niger and Bani Rivers and watersheds and (*b*) an example of individual sites in the irrigated agricultural system for Pont Gendarme in Senegal.

The study was conducted in two major sampling events in February and March (winter) of 2011 and September and October (autumn) of 2011, at irrigated agricultural sites in six countries. In winter 2011, we sampled in three countries at seven agricultural locations/cities: Pont Gendarme and Dagana in Senegal; Kaedi, Bogue and Rosso in Mauritania; and Selingue and Kayes in Mali (figure 2*a*). Two to six samples were collected in the irrigation water system at each location/city (later referred to as ‘field campaign’). In autumn 2011, we sampled in four countries at nine agricultural locations/cities: Kankan and Siguiri, Guinea; Gaya, Say and Toula, Niger; Diola and Niona, Mali; Malanville, Benin; and again at Dagana, Senegal. Three to six samples were collected in the irrigated water system at each agricultural location. Table 1 lists individual site descriptions, abbreviations, country, field campaign name, deployment dates and latitude and longitude. Figure 2 shows all field campaigns along the Niger, Senegal and Bani Rivers and watersheds. Figure 2*b* depicts individual sites in the agricultural location of Pont Gendarme, Senegal and provides an example of the irrigated agricultural systems in this region. As presented in table 1, the agricultural systems consist of engineered irrigation systems. PSDs were deployed in the water of the canals and drainage areas of these systems.

**Table 1.** Field sampling locations, site names, abbreviations, latitude and longitude in decimal degrees (dd), country, field campaign name and PSD deployment dates for winter and autumn 2011.

country	field campaign	site name	latitude (dd)	longitude (dd)	sampling dates
Senegal	Pont Gendarme	main drainage	16.24209	−16.21390	9 Feb–23 Feb
		drainage	16.24311	−16.23215	
		irrigation	16.25432	−16.20718	
		river irrigation	16.26234	−16.21101	
		low base weir	16.23038	−16.20931	
Senegal	Dagana	drainage	16.56984	−15.47515	8 Feb–22 Feb
		irrigation I	16.57652	−15.48223	
		irrigation II	16.56579	−15.48393	
		main drainage	16.57628	−15.48207	
		upstream pump	16.57665	−15.48226	
		low base weir	16.57366	−15.48215	
Mauritania	Kaedi	prepump	16.14878	−13.48697	12 Mar–26 Mar
		postpump	16.14870	−13.48628	
		irrigation	16.14227	−13.46677	
Mauritania	Bogue	pump	16.58668	−14.26808	12 Mar–25 Mar
		prepump	16.58665	−14.28435	
		postpump	16.58567	−14.27980	
Mauritania	Rosso	prepump	16.51757	−15.89287	10 Mar–24 Mar
		postpump	16.50722	−15.82550	
		drainage I	16.51595	−15.86725	
		drainage II	16.50722	−15.82277	
		irrigation	16.51643	−15.86767	
		pump	16.51613	−15.86773	
Mali	Selingue	drainage	11.72542	−8.20175	10 Feb–24 Feb
		main drainage	11.69912	−8.22107	
		irrigation I	11.68773	−8.21475	
		irrigation II	11.64718	−8.21630	
Mali	Kayes	river water	14.45755	−11.45667	8 Feb–22 Feb
		irrigation	14.40725	−11.37727	
Senegal	Dagana	drainage	16.56984	−15.47515	15 Sept–29 Sept
		irrigation I	16.57652	−15.48223	
		irrigation II	16.56579	−15.48393	
		main drainage	16.57628	−15.48207	
		upstream pump	16.57665	−15.48226	
Benin	Malanville	pump	11.88278	3.35353	20 Sept–5 Oct
		drainage	11.87808	3.39433	
		lowland I	11.86964	3.37364	
		lowland II	11.88506	3.36003	
Mali	Diola	river	12.65978	−6.54265	15 Sept–29 Sept
		drainage I	12.65720	−6.54340	
		drainage II	12.67153	−6.53413	
Mali	Niono	outlet pump	14.24518	−5.99425	13 Sept–27 Sept
		irrigation	14.26190	−5.92165	
		river	14.22920	−5.99187	
Niger	Gaya	drainage	n.a.	n.a.	20 Sept–5 Oct
		irrigation reservoir			
		pump			
Niger	Say	irrigation I	n.a.	n.a.	14 Sept–20 Sept
		irrigation II			
		pump I			
		pump II			
		drainage			
Niger	Toula	drainage	n.a.	n.a.	13 Sept–28 Sept
		main drainage			
		pump			
		irrigation I			
		irrigation II			
Guinea	Kankan	Niger river	11.06836	−9.22717	20 Sept–04 Oct
		Milo river	11.06083	−9.22208	
		drainage	11.05194	−9.24197	
Guinea	Siguiri	Niger river	11.40701	−9.08665	19 Sept–03 Oct
		drainage I	11.41010	−9.15726	
		drainage II	11.40447	−9.19083

#### (ii) Sampling

Over the course of this study, 16 different sampling events were conducted (field sampling dates listed in table 1). We collected a total of 63 field samples and 16 field QC samples. All samples were collected as matched sets that were then allocated to CERES and OSU (table 1). Stainless steel deployment cages, manufactured in Senegal, were deployed in the water column as described elsewhere [13]. Following each nominal 14-day deployment, CERES personnel retrieved samplers from the field. One sampler from each pair was shipped to OSU and the other was analysed at CERES. Each laboratory cleaned PSDs as previously described [7,24] and stored them at −20°C in glass jars until analysis. Samplers were spiked with tetrachloro-meta-xylene (TCMX) and PCB-209 as surrogates prior to extraction, then extracted by dialysis in *n*-hexane as previously described [23].

### (d) Chemicals

The following pesticides and related compounds were analysed in PSD samples: alachlor, aldrin, bifenthrin, α-BHC, β-BHC, δ-BHC, γ-BHC (lindane), captafol, captan, chlorobenzilate, *cis*-chlordane (α-chlordane), *trans*-chlordane (γ-chlordane), chloroneb, chlorpyrifos, chlorothalonil, dacthal, diallate, dieldrin, dimethoate, 4,4′-DDD, 4,4′-DDE, 4,4′-DDT, endosulfan sulfate, endosulfan I, endosulfan II, endrin, endrin aldehyde, endrin ketone, esfenvalerate, heptachlor, heptachlor epoxide, hexachlorocyclopentadiene (HCCPD), hexachlorobenzene, isodrin, methoxychlor, metolachlor, mirex, *trans*-nonachlor, propachlor, prophos, *cis*-permethrin, *trans*-permethrin, terrazole and trifluralin. PCB-100, PCB-180 and pentachloronitrobenzene were from Cambridge Isotope Laboratories (Andover, MA). TCMX and PCB-209 were used as sample preparation performance surrogates, and 4,4′-dibromooctafluorobiphenyl was used as an internal standard. Native pesticides, surrogates and internal standards were supplied by AccuStandard (New Haven, CT).

### (e) Quantitative chemical analysis and de-convolution reporting software screening

PSD extracts were analysed for 44 compounds including PRCs, internal standards and internal surrogates using an Agilent 6890N gas chromatograph (GC) with dual 7683 injectors, dual columns and dual electron capture detectors (ECD). The GC was configured with Agilent DB-XLB and Agilent DB-17MS columns. In addition to quantitative evaluation, OSU extracts were also screened for the presence/absence of over 1100 chemicals of concern (see electronic supplementary material, table S2) on an Agilent 6890N GC with 5975B Mass Selective Detector with a DB-5MS column. Limits of quantitation (LOQ) are detailed in the electronic supplementary material.

### (f) Quality control

There were over 125 QC samples generated and analysed during the capacity-building and monitoring study. QC samples included field and trip blanks for each deployment/retrieval, PSD fabrication blanks, laboratory preparation blanks, reagent blanks, instrument blanks, continuing calibration verification (CCV) and spikes. In the paired study, all target compounds were below the level of quantitation in all blank QC samples, and recoveries of CCV and spikes were within the data quality objectives (DQOs) of ±20% of true value.

### (g) Water concentration determination

Water concentrations were calculated using the empirical uptake model with PRC-derived sampling rates [7,22,23,25]. The equations used to calculate the water concentrations presented in this study are detailed elsewhere [23]. This model is based on uptake kinetics and does not require assumptions about individual analytes being at equilibrium or in the linear uptake range. The use of PRCs allows for an accurate determination of *in situ*, site-specific sampling rates under variable exposure conditions, including variable temperatures, flow rates and biofouling [22,23]. Additionally, it is not necessary for the analytes of interest to reach equilibrium with the sampler to determine sampling rates [7]; therefore, variable sampling deployment times are comparable. PCBs 100 and 180 and pentachloronitrobenzene were used as the PRCs for the calculations. These PRCs cover a range of log *K*_ow_ values that makes them adequate for deriving the uptake rates of the pesticide analytes included in this study [22]. When PRC recoveries were below 20% or above 80%, the sampling rates were determined using an improved model for calculating *in situ* sampling rates [21,23].

Approximate 95% confidence intervals (CIs) of mean *C*_free_ pesticide concentrations were determined using methods previously described for PSDs, where log_10_ measurement variances display approximately normal distributions [26]. Specific details of the calculation are provided in the electronic supplementary material. As a result of scale transformation, approximate intervals are asymmetric on the measurement scale used**.**

## 3. Results and discussion

### (a) Capacity building

#### (i) Pesticide analytical skill and knowledge training

**Figure 3.**
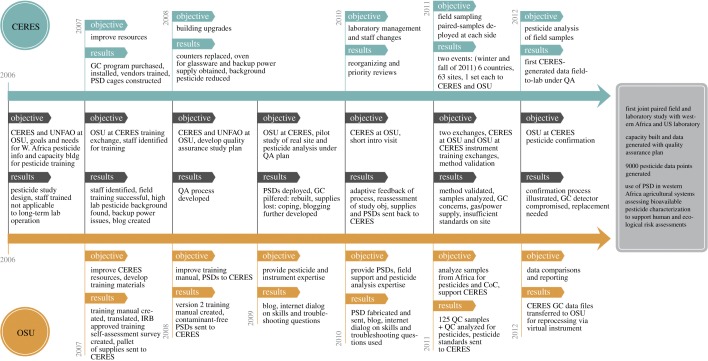
Timeline of objectives and results of the capacity-building events: delineates the joint and individual activities by CERES and OSU. The project culminated in a field and laboratory study where paired PSD samples were simultaneously analysed in African and US laboratories with QC evaluation and traceability. The joint study included sampling from 63 sites across six West African countries, generating a 9000 data point pesticide database with virtual access to all study participants. CoC, chain of custody.

Pesticide capacity ‘fit-for-purpose’ for environmental monitoring was identified through the joint goals and assessments process and further confirmed and refined through CERES scientist self-assessments. Figure 3 delineates the dynamic flow of the project with a timeline of event objectives and results of the partnership activities during various phases of the capacity-building programme. Figure 1 summarizes the pesticide monitoring capacity-building challenges, strategies, methods and outcomes. The intent was to build staff technical capacity from a foundation provided by their preparatory chemistry courses, combined with analytical chemistry work experience. Ten CERES staff received PSD pesticide training. Often identifying the correct staff to receive training and retention is problematic in capacity-building endeavours. In this study, half of the staff participated in multiple training events. Importantly, half of the staff trained was retained by the programme for over 2 years. Early in the collaboration, training manuals and 26 SOPs (English and French versions) were exchanged along with three training videos. We found the application of several pedagogical principles maximized training and learning: repetition to develop lasting rigorous skill sets, hands-on training at every level, mentoring and delivering training in discrete modules. It became apparent that trying to both train and learn all aspects (e.g. QA, field skills, advanced laboratory skills for trace pesticide residues, PSDs, gas chromatography, etc.) could not effectively be completed in a single training event. A more effective method used a modular approach, which built on previous training sets and worked to correct any lingering issues from previous training events. From these eight joint events held throughout the project effective gain resulted by integrating members from CERES and OSU through cycling training sites between US and West Africa, enabling the trainer/trainee to be both host and visitor.

Initially, CERES staff benefited from advanced training that encompassed all aspects of analytical chemistry including, but not limited to, sample preparation, analysis, instrument maintenance, record keeping and laboratory safety. The results of the CERES staff self-evaluation support this observation. Their average general self-assessment prior to training was 3 and after training above 4. However, both the trainer and self-assessment suggested additional analytical technique training was warranted to continue to improve skills.

The most challenging component of the study was instrument operation and data analysis. The programme contributed a GC with dual ECDs, which has excellent sensitivity for detecting pesticides and requires less gas and consumables than other analytical platforms. These resources are often difficult to acquire in Senegal. Additionally, the overall maintenance of the GC-ECD is typically less technically advanced than other analytical instruments. Initially, chromatography skills were insufficient to manage CERES's laboratory equipment and analysis. Though major improvements were made, maintaining adequate supplies for the instruments and developing expertise for routine instrument maintenance continue to challenge the programme.

#### (ii) Improving and integrating quality assurance

Field and laboratory QC documents were created for the project. All field QC met DQOs. Nearly all in-laboratory QC met DQOs by the end of the study period. The CERES staff quickly learned the QA procedures although some aspects were initially more demanding than existing procedures at CERES. No established criterion for method validation or demonstration initially existed at CERES. The PSD pesticide method was validated as part of the 2011 OSU–CERES training exchange. We recognize that the demonstration of ability and traceability are critical to successfully developing a monitoring system for the effects of pesticides on human and environmental health in West Africa. Lack of sufficient staff with pesticide expertise remained a challenge and manifested itself in many ways that challenged capacity building. One example is that CERES cannot consistently apply peer or senior review because often qualified staff is not available to provide this critical service.

#### (iii) Resources

We found during the first OSU training at CERES that the laboratory facilities were inadequate for trace pesticide analysis (figures 1 and 3). Contamination of the laboratory and equipment was one early problem. The project overcame some of these inadequacies through remodelling the tile/grout counters with stainless steel and acquiring a power supply backup and an oven to bake-out glassware. Isolating some equipment, such as pipettes, that were used at CERES for pesticide formulation analysis, which was concurrent in the facility, also helped to reduce laboratory contamination. However, some facility and resource challenges, for example, power supply irregularities, persisted.

#### (iv) Passive sampling devices training

CERES staff excelled at PSD fabrication. Ten staff members were trained in all aspects of PSD including fabrication, spiking, field use and laboratory extraction (figure 1). The PSD QC from each step met DQOs. PSDs are uniquely simple to fabricate and use. PSD deployment and retrieval in the field was an unqualified success and each field training session with staff was also successful. Despite some staff turnover at CERES, fieldwork remained successful. The project was able to retain institutional knowledge and continued training exchanges often included refresher field training. The results of the CERES staff self-evaluation compare well with this assessment. Skill levels on self-assessments prior to PSD training were on average 2.5 and 6 after training (scale 1–7). Observations of OSU trainers support these self-assessment results and complete questionnaire results are provided in the electronic supplementary material.

### (b) Data sharing and quality control comparisons between laboratories

A general data management strategy was established to compare data between laboratories. OSU uses a commercial laboratory information management system (LIMS) providing sample indexing, workflow management, QA document generation and automation of data collection, approval and reporting. The sample data and results from both laboratories were stored in LIMS. Inherent difficulties arose in providing remote access to the LIMS web interface to users at the CERES laboratory in Dakar, Senegal. To overcome this obstacle, a pilot study employed a virtual instrument deployed outside the OSU firewall but on the LIMS system domain and enabled LIMS access remotely while circumventing the firewall. Dropbox [27] was used as an intermediary to move raw instrument data and final results from the CERES laboratory to the virtual instrument at OSU. Although the remote desktop connection latency was problematic for operating the LIMS web interface remotely from CERES, the review of chromatographic data generated at CERES and troubleshooting data analysis from OSU was a success. The secure remote access of data is detailed in the electronic supplementary material.

Three QC datasets are presented, one set is from PSDs extracted, analysed and data processed by CERES, called ‘CERES data’. The next set is from PSDs extracted and analysed by CERES, but where the raw data from the CERES GC were reprocessed by OSU staff. This dataset is called ‘OSU-reprocessed CERES data’. The final set is from samples transported to OSU and analysed at OSU. The dataset is called ‘OSU data’ (table 2). The CERES results show that nearly all pesticides were found in nearly all samples. This was owing to an inadequate understanding of the confirmation process required when using the dual column, dual detector GC-ECD pesticide method. The process of confirmation includes identifying the target analytes by comparing the retention time of the peak on both columns of the GC-ECD as well as other data elements, described in more detail in the electronic supplementary material. The CERES analysis of data did not adequately employ this confirmation process, leading CERES staff to report analytes as present even when they were not confirmed. When OSU reprocessed CERES data using the confirmation process most blank QC samples were below the level of quantitation. Although the detection limits for the CERES data are higher than OSU's detection limits (*data not shown*), there was good agreement between the OSU-reprocessed CERES data and OSU data for field and trip blanks. The performance of CCV standards indicated that CERES’ instrument was functioning adequately at the time of sample analysis and training, where most QC recoveries were between 75 and 125% of the true value. The other QC samples, instrument blanks, spikes, laboratory preparation blanks, reagent blanks and cleaning blanks were within the DQOs defined for the programme. This demonstrates that CERES is technically proficient in the construction and handling of PSDs, but that additional training and practice are necessary for instrument operation and data processing.

**Table 2.** Comparison of QC samples between CERES-generated data, OSU processing of CERES data and OSU-generated datasets. QC summary (ng ml^−1^); BLOD, below limit of detection; CCV, continuing calibration verification; n.a., not applicable; s.d., standard deviation.

compound list	CERES data				OSU reprocessed CERES data								
	trip blanks, *n* = 7		field blanks, *n* = 7		instrument blanks, *n* = 4		CCVs, *n* = 4			trip blanks, *n* = 7		field blanks, *n* = 7	
	average	s.d.	average	s.d.	average	s.d.	average	s.d.	average recovery (%)	average	s.d.	average	s.d.
4,4′-DDD	259	671	2526	6499	BLOD	n.a.	434	50	87	BLOD	n.a.	BLOD	n.a.
4,4’-DDE	2	2	31	66	BLOD	n.a.	573	69	115	BLOD	n.a.	BLOD	n.a.
4,4’-DDT	11	25	41	87	BLOD	n.a.	481	124	96	BLOD	n.a.	BLOD	n.a.
alachlor	118	269	44 532	11 6474	BLOD	n.a.	377	56	75	BLOD	n.a.	BLOD	n.a.
aldrin	5	4	28	47	BLOD	n.a.	513	73	103	BLOD	n.a.	BLOD	n.a.
α-BHC	1426	1972	1270	1601	BLOD	n.a.	1385	26	277	BLOD	n.a.	BLOD	n.a.
α-chlordane	1	1	858	2171	BLOD	n.a.	400	33	80	BLOD	n.a.	BLOD	n.a.
β-BHC	65	166	115	253	BLOD	n.a.	385	26	77	BLOD	n.a.	BLOD	n.a.
captan	4860	7797	2487	2551	BLOD	n.a.	457	52	91	BLOD	n.a.	BLOD	n.a.
chlorobenzilate	52 580 919	139 087 237	1391	1687	BLOD	n.a.	470	134	94	BLOD	n.a.	BLOD	n.a.
chloroneb	574	1386	580	839	BLOD	n.a.	394	70	79	BLOD	n.a.	BLOD	n.a.
chlorothalonil	10 134	12 899	58 620	153 231	BLOD	n.a.	508	39	102	BLOD	n.a.	BLOD	n.a.
chlorpyrifos	3	7	76	84	BLOD	n.a.	194	227	39	BLOD	n.a.	BLOD	n.a.
*cis*-permethrin	2701	6905	2254	4281	BLOD	n.a.	391	32	78	BLOD	n.a.	BLOD	n.a.
dacthal	16	41	23	42	BLOD	n.a.	221	256	44	BLOD	n.a.	BLOD	n.a.
dieldrin	28	73	33 453	75 018	BLOD	n.a.	511	17	102	BLOD	n.a.	BLOD	n.a.
dimethoate	153	133	238	354	BLOD	n.a.	139	278	28	BLOD	n.a.	BLOD	n.a.
endosulfan I	8	18	98	175	BLOD	n.a.	427	37	85	BLOD	n.a.	BLOD	n.a.
endosulfan II	3	3	60	83	BLOD	n.a.	549	73	110	BLOD	n.a.	BLOD	n.a.
endosulfan sulfate	3905	10 258	8113	15 905	BLOD	n.a.	459	67	92	BLOD	n.a.	BLOD	n.a.
endrin	17	37	263	482	BLOD	n.a.	442	22	88	BLOD	n.a.	BLOD	n.a.
endrin aldehyde	4505	8025	12 267	19 902	BLOD	n.a.	484	9	97	BLOD	n.a.	BLOD	n.a.
endrin ketone	34	47	72	85	BLOD	n.a.	472	64	94	BLOD	n.a.	BLOD	n.a.
esfenvalerate	1350	3488	859	1471	BLOD	n.a.	294	143	59	BLOD	n.a.	BLOD	n.a.
γ-chlordane	593	1303	4047	6002	BLOD	n.a.	1775	1286	355	BLOD	n.a.	BLOD	n.a.
HCCPD	2668	6975	1283	1944	BLOD	n.a.	412	299	82	BLOD	n.a.	BLOD	n.a.
heptachlor	269	689	193	212	BLOD	n.a.	390	36	78	BLOD	n.a.	BLOD	n.a.
heptachlor epoxide	90	236	51	68	BLOD	n.a.	491	51	98	BLOD	n.a.	BLOD	n.a.
hexachlorobenzene	731	988	1362	1659	BLOD	n.a.	452	66	90	BLOD	n.a.	BLOD	n.a.
isodrin	1	1	31	41	BLOD	n.a.	475	40	95	BLOD	n.a.	BLOD	n.a.
lindane	6	11	13	23	BLOD	n.a.	412	38	82	BLOD	n.a.	BLOD	n.a.
methoxychlor	349	518	780	1521	BLOD	n.a.	291	198	58	BLOD	n.a.	BLOD	n.a.
metolachlor	537	1404	421	636	BLOD	n.a.	488	74	98	BLOD	n.a.	BLOD	n.a.
mirex	1944	5001	1148	1935	BLOD	n.a.	477	10	95	BLOD	n.a.	BLOD	n.a.
prophos	1236	3121	1040	2245	BLOD	n.a.	486	69	97	BLOD	n.a.	BLOD	n.a.
terrazole	33	63	121	263	BLOD	n.a.	482	117	96	BLOD	n.a.	BLOD	n.a.
*trans*-permethrin	4041	10 374	1919	3045	BLOD	n.a.	446	50	89	BLOD	n.a.	BLOD	n.a.
trifluralin	341	827	323	723	BLOD	n.a.	442	102	88	BLOD	n.a.	BLOD	n.a.

	OSU data														
	instrument blanks, *n* = 10		CCVs, *n* = 10			trip blanks, *n* = 7		field blanks, *n* = 7		spikes, *n* = 4		lab prep blanks, *n* = 8		extraction blank, *n* = 1	post deployment cleaning blank, *n* = 1
	average	s.d.	average	s.d.	average recovery (%)	average	s.d.	average	s.d.	average recovery (%)	s.d.	average	s.d.		
4,4′-DDD	BLOD	n.a.	555	31	111	BLOD	n.a.	BLOD	n.a.	130	10	BLOD	n.a.	BLOD	BLOD
4,4’-DDE	BLOD	n.a.	538	22	108	BLOD	n.a.	BLOD	n.a.	114	8	BLOD	n.a.	BLOD	BLOD
4,4’-DDT	BLOD	n.a.	535	30	107	BLOD	n.a.	BLOD	n.a.	98	12	BLOD	n.a.	BLOD	BLOD
alachlor	BLOD	n.a.	517	28	103	BLOD	n.a.	BLOD	n.a.	113	7	BLOD	n.a.	BLOD	BLOD
aldrin	BLOD	n.a.	534	15	107	BLOD	n.a.	BLOD	n.a.	108	5	BLOD	n.a.	BLOD	BLOD
α-BHC	BLOD	n.a.	525	14	105	BLOD	n.a.	BLOD	n.a.	109	8	BLOD	n.a.	BLOD	BLOD
α-chlordane	BLOD	n.a.	532	18	106	BLOD	n.a.	BLOD	n.a.	115	9	BLOD	n.a.	BLOD	BLOD
β-BHC	BLOD	n.a.	567	28	113	BLOD	n.a.	BLOD	n.a.	112	3	BLOD	n.a.	BLOD	BLOD
captan	BLOD	n.a.	399	100	80	BLOD	n.a.	BLOD	n.a.	BLOD	n.a.	BLOD	n.a.	BLOD	BLOD
chlorobenzilate	BLOD	n.a.	440	53	88	BLOD	n.a.	BLOD	n.a.	84	54	BLOD	n.a.	BLOD	BLOD
chloroneb	BLOD	n.a.	527	28	105	BLOD	n.a.	BLOD	n.a.	100	8	BLOD	n.a.	BLOD	BLOD
chlorothalonil	BLOD	n.a.	538	49	108	BLOD	n.a.	BLOD	n.a.	78	12	BLOD	n.a.	BLOD	BLOD
chlorpyrifos	BLOD	n.a.	545	15	109	BLOD	n.a.	BLOD	n.a.	151	12	BLOD	n.a.	BLOD	BLOD
*cis*-permethrin	BLOD	n.a.	552	27	110	BLOD	n.a.	BLOD	n.a.	125	22	BLOD	n.a.	BLOD	BLOD
dacthal	BLOD	n.a.	532	19	106	BLOD	n.a.	BLOD	n.a.	112	6	BLOD	n.a.	BLOD	BLOD
dieldrin	BLOD	n.a.	504	49	101	BLOD	n.a.	BLOD	n.a.	82	13	BLOD	n.a.	BLOD	BLOD
dimethoate	BLOD	n.a.	633	110	127	BLOD	n.a.	BLOD	n.a.	265	67	BLOD	n.a.	BLOD	BLOD
endosulfan I	BLOD	n.a.	538	25	108	BLOD	n.a.	BLOD	n.a.	130	15	BLOD	n.a.	BLOD	BLOD
endosulfan II	BLOD	n.a.	557	34	111	BLOD	n.a.	BLOD	n.a.	150	24	BLOD	n.a.	BLOD	BLOD
endosulfan sulfate	BLOD	n.a.	483	30	97	BLOD	n.a.	BLOD	n.a.	124	30	BLOD	n.a.	BLOD	BLOD
endrin	BLOD	n.a.	1215	425	243	BLOD	n.a.	BLOD	n.a.	207	43	BLOD	n.a.	BLOD	BLOD
endrin aldehyde	BLOD	n.a.	574	38	115	BLOD	n.a.	BLOD	n.a.	97	17	BLOD	n.a.	BLOD	BLOD
endrin ketone	BLOD	n.a.	549	51	110	BLOD	n.a.	BLOD	n.a.	108	5	BLOD	n.a.	BLOD	BLOD
esfenvalerate	BLOD	n.a.	526	331	105	BLOD	n.a.	BLOD	n.a.	4	8	BLOD	n.a.	BLOD	BLOD
γ-chlordane	BLOD	n.a.	557	30	111	BLOD	n.a.	BLOD	n.a.	108	7	BLOD	n.a.	BLOD	BLOD
HCCPD	BLOD	n.a.	450	81	90	BLOD	n.a.	BLOD	n.a.	BLOD	n.a.	BLOD	n.a.	BLOD	BLOD
heptachlor	BLOD	n.a.	538	49	108	BLOD	n.a.	BLOD	n.a.	96	95	BLOD	n.a.	BLOD	BLOD
heptachlor epoxide	BLOD	n.a.	534	22	107	BLOD	n.a.	BLOD	n.a.	118	8	BLOD	n.a.	BLOD	BLOD
hexachlorobenzene	BLOD	n.a.	522	12	104	BLOD	n.a.	BLOD	n.a.	114	9	BLOD	n.a.	BLOD	BLOD
isodrin	BLOD	n.a.	523	20	105	BLOD	n.a.	BLOD	n.a.	96	6	BLOD	n.a.	BLOD	BLOD
lindane	BLOD	n.a.	520	7	104	BLOD	n.a.	BLOD	n.a.	115	7	BLOD	n.a.	BLOD	BLOD
methoxychlor	BLOD	n.a.	1155	380	231	BLOD	n.a.	BLOD	n.a.	106	10	BLOD	n.a.	BLOD	BLOD
metolachlor	BLOD	n.a.	540	19	108	BLOD	n.a.	BLOD	n.a.	97	21	BLOD	n.a.	BLOD	BLOD
mirex	BLOD	n.a.	522	24	104	BLOD	n.a.	BLOD	n.a.	101	17	BLOD	n.a.	BLOD	BLOD
prophos	BLOD	n.a.	513	35	103	BLOD	n.a.	BLOD	n.a.	116	5	BLOD	n.a.	BLOD	BLOD
terrazole	BLOD	n.a.	539	24	108	BLOD	n.a.	BLOD	n.a.	116	9	BLOD	n.a.	BLOD	BLOD
*trans*-permethrin	BLOD	n.a.	551	31	110	BLOD	n.a.	BLOD	n.a.	117	14	BLOD	n.a.	BLOD	BLOD
trifluralin	BLOD	n.a.	509	20	102	BLOD	n.a.	BLOD	n.a.	124	10	BLOD	n.a.	BLOD	BLOD

The sensitivity of the CERES GC was significantly less than OSU's instrument. The lack of quality instrument sensitivity was likely owing to the subsequent identification of inadequate gas quality from a poorly maintained gas generator used for the GC. Gas quality is known to impact the sensitivity of GC detectors. Electrical challenges at the CERES laboratory likely led to detector degradation as well. Owing to the lack of sensitivity, the CERES dataset was not adequate to make any rigorous comparisons with the OSU dataset.

### (c) Chemical characterization

PSDs were used to quantify the bioavailable fraction of contaminants of concern in agricultural systems in Mali, Mauritania, Guinea, Niger, Benin and Senegal. Concentrations in the ng l^−1^ or parts per trillion (ppt) range may seem low; however, the methodology used in this study only measures freely dissolved chemicals (*C*_free_) in the water column, which does not include suspended, particulate-bound or any other undissolved fraction. The reported concentrations are representative of what an organism in the water column could absorb through passive partitioning, which is the dominant route of uptake for fish and shellfish [11,12]. The individual site data presented here are exclusively from the OSU extraction and analysis datasets (see electronic supplementary material, table S3). The CERES-analysed dataset was not comparable owing to improper confirmation procedures used at CERES; the OSU-reprocessed CERES data were not fit for comparisons owing to poor recoveries and higher levels of quantitation. These outcomes are discussed in detail below.

**Figure 4.**
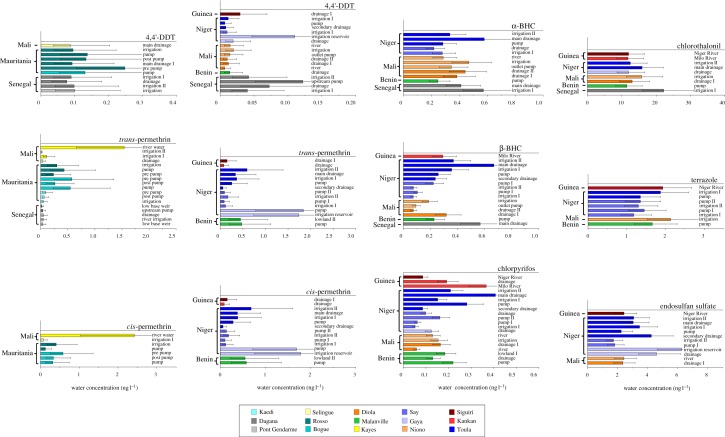
Select bioavailable (*C*_free_) pesticide concentrations in: Mali, Mauritania, Senegal, Guinea, Niger and Benin with individual locations/sites indicated by colour code, see legend. Sites not included in the graphs were below the level of quantitation for the contaminant.

The most abundant pesticides during the winter 2011 sampling in Senegal, Mauritania and Mali, were 4,4′-DDT and the *cis*- and *trans*-permethrins (figure 4). Sites not displayed in the individual pesticide figures were below our level of quantitation (see electronic supplementary material, table S3). When present, the dissolved 4,4′-DDT levels in Mali, Mauritania and Senegal in winter 2011 ranged from 0.1 to 0.2 ppt. 4,4′-DDT (and 4,4′-DDD and 4,4′-DDE data not shown) was found at many sites in Guinea, Niger, Mali, Benin and Senegal in the autumn 2011 sampling event. The 4,4′-DDT bioavailable concentrations typically ranged from 0.01 to 0.15 ppt in autumn of 2011. The highest concentrations of 4,4′-DDT/DDD/DDE (0.1 and 0.2 ppt) were at the drainage 1 site at Siguiri, Guinea and irrigation reservoir site at Gaya, Niger, respectively. The 4,4′-DDT was generally higher in the winter sampling than the autumn sampling. The Dagana, Senegal site was sampled in both winter and autumn and river flow and cropping practices differed between the events. At Dagana, 4,4′-DDT was found during both sampling events, likely owing to its environmental persistence. The Dagana site did not differ significantly between winter and autumn sampling events, measuring 0.1 ppt and 0.05–0.1 ppt, respectively. The US Environmental Protection Agency (USEPA) estimates that the maximum concentration of 1 ng l^−1^ (ppt) of total 4,4′-DDT and metabolites in water yields protection of aquatic life from potential exposure-related effects [28,29]. This is about 10-fold greater than the levels we measured.

The bioavailable *cis*- and *trans*-permethrins ranged from about 0.1 to 2.5 ppt in the winter 2011 sampling. The highest concentrations of permethrins were measured in Mali, at the river water site. We observed that many of the Mauritania sites had permethrins present, in the 0.1–1 ppt range. Permethrins were also found in the autumn 2011 sampling. Sites in Guinea, Niger and Benin had 0.1–2 ppt *cis*- and *trans*-permethrins. The pump and irrigation reservoir sites in Gaya, Niger had the highest measured concentrations. We recognize that *cis*- and *trans*-permethrins are often used as insect repellents and sometimes to treat clothing. While this represents a potential source of cross contamination, we did not find any consistent presence in the field, trip or reagent blanks collected and analysed throughout the study with the OSU datasets.

Permethrins are extremely toxic to fish and other aquatic life, so it is noteworthy when they are used near water sources or found in water systems as demonstrated here [30,31]. The toxicity is believed to occur primarily from the dissolved, bioavailable fraction, not from the particulate-bound fraction [32–34]. Because PSD technology measures *C*_free_, the levels reported are meaningful. A draft chronic criterion for water toxicity has been described and proposed at 2 ng l^−1^ (ppt) permethrins, which is a level similar to those measured in this study [35]. Permethrins have also been identified as a potential threat to beneficial insects, including honeybees [36]. However, a risk/benefit balance analysis would also recognize permethrins offer substantial benefits to users in agriculture and public health for mosquito control.

α-BHC (1,2,3,4,5,6,-hexachlorocyclohexane, α-HCH) and β-BHC, both isomers of hexachlorocyclohexane (HCH) part of the lindane insecticide production, were found at many sites in Niger and Mali, and at some sites in Guinea, Benin and Senegal (figure 4). The active ingredient γ-BHC was found occasionally but was below the level of quantitation. This is to be expected as the inert lindane isomers, such as α- and β-BHC are known to be more persistent in soils [37]. The USEPA estimates that concentrations less than 2.6 and 9.1 ng l^−1^, for α-BHC and β-BHC and metabolites, respectively, will provide maximum protection for human health from potential carcinogenic effects associated with the ingestion of contaminated water and aquatic organisms [28,29]. The *C*_free_ found were typically in the 0.2–0.5 ppt range, about a factor of fivefold to 10-fold below the USEPA recommended water quality criterion. The highest values measured were at the main drainage site in Toula, Niger, 0.6 and 0.7 ppt for α- and β-BHC, respectively.

Chlorpyrifos was found at many sites in Guinea, Niger, Mali and Benin, from 0.1 to 0.4 ppt. The highest levels were observed at Milo River Kankan, Guinea and the main drainage Toula, Niger. Sites not included in the graphs in figure 4 were below our level of quantitation (see electronic supplementary material, table S3). Chlorothalonil was found at a few sites, typically 10–20 ppt when detected. Similarly, terrazole was also found at a few sites (1–2 ppt). Neither chlorothalonil nor terrazole was reportedly used during the survey conducted concurrent with this study [18]. Their presence at these few sites could be the result of legacy or undocumented uses. Endosulfan I, II and endosulfan sulfate were measured at 0.3–1, 0.5–1.5 and 2–4 ppt, respectively, when present above our LOQ. Owing to the similar toxicity of all individual endosulfans, the guidelines are typically represented as the sum. The sum of endosulfans ranged from 1 to 11 ppt in our study.

The PSDs used in this study are designed for semi-polar and non-polar organic compounds, such as contaminants with octanol-water partition coefficients (log *K*_ow_) > 3.0. Although not optimized for polar compounds, we sequestered and confirmed more polar contaminants, for example dimethoate (log *K*_ow_ = 0.7), at some sites in the ppb range, despite dimethoate's relatively rapid degradation. This is consistent with Jepson *et al.* [18] survey results of pesticide use where dimethoate was reported as one of the more heavily used pesticides. Future scheduled research will couple two complementary passive sampling materials: the polyethylene used in this study and silicone-based polymers optimized to sequester more polar contaminants. Dacthal was also measured at a few sites, notably Senegal irrigation 1 site at 5 ppt, as well as other sites in Guinea, Niger and Benin all at 1 ppt or less.

Because PSDs provide the bioavailable fraction, *C*_free_, the resulting concentrations are directly related to risk. The water at these sites is used not only for agriculture but also for drinking, bathing, washing dishes and clothing and aquaculture. Measured concentrations are bioavailable to resident fish, and to the people who will likely eat those fish, as well as bathe in and drink the water, warranting further human and ecological health risk assessments. Exposure and risk criteria should take into consideration the protection of human health, in addition to the protection of aquatic organisms and ecosystems.

**Figure 5.**
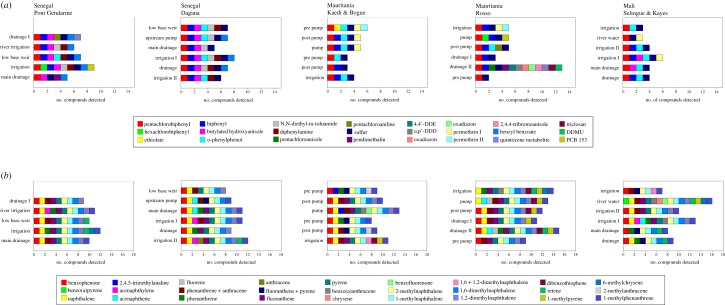
(*a*,*b*) Detection of chemical of concern identified in samples using mass spectral de-convolution and identification in PSD extracts from Africa. (*a*,*b*) The number of chemicals of concern that were identified in samples from each site. The colours of the blocks indicate which chemicals were identified in the sample. (*a*) PCBs and pesticides and (*b*) PAHs and substituted PAHs.

Screening environmental samples for the presence/absence of over 1100 chemicals of concern identified 10–28 chemicals beyond the target pesticides (figure 5). This level of resolution in the chemical characterization of environmental samples lends significant additional depth to the assessment of water quality. Chemicals identified with DRS included PCBs, herbicides, insecticides, PAHs and substituted PAHs. In addition to these compounds, substances from personal care products were identified. Triclosan, an antibacterial and antifungal compound, was found at all sites in Senegal as well as several other sites. Musks found in some personal care products (tonalide and traseolide) were detected in many samples (*data not shown*) at Ponte Gendarme, Bogue and Rosso.

The presence of chemicals in samples from western Africa showed spatial variability (figures 4 and 5). While a greater number of chemicals of concern were detected in samples obtained from the drainage II site at Rosso, Mauritania, the fewest chemicals were detected at the prepump site at Rosso, Mauritania. Several more pesticides were detected at many of the Senegal sites (figure 5) and overall fewer pesticides were detected at the Mauritania and Mali sites, with the exception of the drainage II site. The numbers of PAHs and substituted PAHs detected were generally similar, although a few more were detected at the Rosso, Mauritania and Selingue and Kayes, Mali sites.

## 4. Conclusion

Using PSDs as a training platform, West African laboratories, scientists and technicians can be prepared to apply rigorous QA standards and perform advanced chemical analyses. The modular training and data sharing platforms can be adopted by other developing or interested laboratories. This study, in conjunction with continued support and collaboration from other institutions, will help CERES develop into a strong scientific resource for West African communities. CERES would benefit from continued advanced training in analytical chemistry including, but not limited to, sample preparation, instrument maintenance, instrument operation, data processing, record keeping and laboratory safety. The lack of adequate laboratory supplies and proper maintenance of equipment and instruments continue to hinder training and technical capacity.

When working on international collaborations, technical integration of data is difficult, but it can be paramount to the success of a project. The data management plan applied to this project involved using commercial and in-house software systems to capture and serve data to downstream modelling and risk assessment systems. A commercial LIMS product was successfully used as the data source to share information with project collaborators and trainees.

PSDs proved to be effective tools for measuring *C*_free_ organic contaminants, including pesticides, in the water of agricultural systems in western Africa. PSDs can be applied to freshwater and marine ecosystems and their robustness is well suited to remote study sites. This study provides important baseline information for assessing the potential risk of exposure to a wide range of chemicals of concern, and the preliminary results indicate a need for further monitoring and risk assessment. Pesticides considered to be obsolete were detected throughout the study area, suggesting that these chemicals are currently being used and/or entering the environment from nearby legacy sources. Additionally, our data demonstrate that emerging contaminants from other anthropogenic sources and personal care products are present. Owing to the difficulty of resolving small differences in pesticides’ concentrations, however, these data should be interpreted with caution. Apparent inconsistencies or trends have yet to be assessed with a rigorous statistical approach. The development of rice and vegetable irrigated lands along the Niger and Senegal Rivers coupled with the potential exposure of local populations to these waters justify the development of more accurate and reliable local methods for the determination of pesticides and contaminants of concern. Reliable pesticide data will be useful information for policymakers, local communities and stakeholders.

## References

[1] Bacci E Calamari D Gaggi C Biney C Focardi S Morosini M 1988 Organochlorine pesticide and PCB residues in plant foliage: (*Mangifera indica*) from West Africa. Chemosphere 17, 693–702. (10.1016/0045-6535(88)90249-4)

[2] Nweke OC Sanders WH III 2009 Modern environmental health hazards: a public health issue of increasing significance in Africa. Environ. Health Perspect 117, 863–870. (10.1289/ehp.0800126) 19590675 PMC2702398

[3] Power AG 2010 Ecosystem services and agriculture: tradeoffs and synergies. Phil. Trans. R. Soc. B 365, 2959–2971. (10.1098/rstb.2010.0143) 20713396 PMC2935121

[4] Alexander M 2000 Aging, bioavailability, and overestimation of risk from environmental pollutants. Environ. Sci. Technol. 34, 4259–4265. (10.1021/es001069+)

[5] Petty JD 2004 A holistic passive integrative sampling approach for assessing the presence and potential impacts of waterborne environmental contaminants. Chemosphere 54, 695–705. (10.1016/j.chemosphere.2003.08.015) 14602102

[6] Gray JS 2002 Perceived and real risks: produced water from oil extraction. Mar. Pollut. Bull. 44, 1171–1172. (10.1016/S0025-326X(02)00357-0) 12523515

[7] Huckins JN Petty JD Booij K 2006 Monitors of organic chemicals in the environment: semipermeable membrane devices. New York, NY: Springer.

[8] Schwartz DA Weis B Wilson SH 2005 The need for exposure health sciences. Environ. Health Perspect. 113, A650 (10.1289/ehp.113-a650) 16203220 PMC1281298

[9] Allan SE Sower GJ Anderson KA 2011 Estimating risk at a superfund site using passive sampling devices as biological surrogates in human health risk models. Chemosphere 85, 920–927. (10.1016/j.chemosphere.2011.06.051) 21741671 PMC3671909

[10] Escher BI Hermens JLM 2004 Internal exposure: linking bioavailability to effects. Environ. Sci. Technol. A, 455A–462A. (10.1021/es0406740) 15597868

[11] Suffet IH Jafvert CT Kukkonen J Servos MR Spacie A Williams LL 1994 Synopsis of discussion session: influences of particulate and dissolved material on the bioavailability of organic compounds. In Bioavailability: physical, chemical, and biological interactions (eds Hamelink JL Landrum PF Bergman HL Benson WH), pp. 93–108. Boca Raton, FL: Lewis Publishers.

[12] Wells JB Lanno RP 2001 Passive sampling devices (PSDs) as biological surrogates for estimating the bioavailability of organic chemicals in soil. In *Environmental toxicology and risk assessment: science, policy, and stadardization–implications for environmental decisions* (eds B Greenberg, R Hull, MH Roberts Jr, RW Gensemer), vol. 10, pp. 253–270. West Conshohocken, PA: ASTM.

[13] Anderson KA Sethajintanin D Sower G Quarles L 2008 Field trial and modeling of uptake rates of in situ lipid free polyethylene membrane passive sampler. Environ. Sci. Technol. 42, 4486–4493. (10.1021/es702657n) 18605575

[14] Adams RG Lohmann R Fernandez LA Macfarlane JK Gschwend PM 2007 Polyethylene devices: passive samplers for measuring dissolved hydrophobic organic compounds in aquatic environments. Environ. Sci. Technol. 41, 1317–1323. (10.1021/es0621593) 17593736

[15] Carls MG Holland LG Short JW Heintz RA Rice SD 2004 Monitoring polynuclear aromatic hydrocarbons in aqueous environments with passive low-density polyethylene membrane devices. Environ. Toxicol. Chem. 23, 1416–1424. (10.1897/03-395) 15376527

[16] Beddington J 2010 Food security: contributions from science to a new and greener revolution. Phil. Trans. R. Soc. B 365, 61–71. (10.1098/rstb.2009.0201) 20008386 PMC2842707

[17] Pretty J 2008 Agricultural sustainability: concepts, principles and evidence. Phil. Trans. R. Soc. B 363, 447–465. (10.1098/rstb.2007.2163) 17652074 PMC2610163

[18] Jepson PC Guzy M Blaustein K Sow M Sarr M Mineau P Kegley S 2014 Measuring pesticide ecological and health risks in West African agriculture to establish an enabling environment for sustainable intensification. Phil. Trans. R. Soc. B 369, 20130491 (10.1098/rstb.2013.0491) 24535399 PMC3928896

[19] Settle W Soumaré M Sarr M Garba MH Poisot A-S 2014 Reducing pesticide risks to farming communities: cotton farmer field schools in Mali. Phil. Trans. R. Soc. B 369, 20120277 (10.1098/rstb.2012.0277) 24535387 PMC3928884

[20] Sower GJ Anderson KA 2008 Spatial and temporal variation of freely dissolved polycyclic aromatic hydrocarbons in an urban river undergoing superfund remediation. Environ. Sci. Technol. 42, 9065–9071. (10.1021/es801286z) 19174872 PMC4172327

[21] Booij K Smedes F 2010 An improved method for estimating in situ sampling rates of nonpolar passive samplers. Environ. Sci. Technol. 44, 6789–6794. (10.1021/es101321v) 20701278

[22] Huckins JN Petty JD Lebo JA Almeida FV Booij K Alvarez DA Cranor WL Clark RC Mogensen BB 2001 Development of the permeability/performance reference compound approach for in situ calibration of semipermeable membrane devices. Environ. Sci. Technol. 36, 85–91. (10.1021/es010991w) 11811495

[23] Allan SE Smith BW Anderson KA 2012 Impact of the deepwater horizon oil spill on bioavailable polycyclic aromatic hydrocarbons in Gulf of Mexico coastal waters. Environ. Sci. Technol. 46, 2033–2039. (10.1021/es202942q) 22321043 PMC3471659

[24] Alvarez DA Petty JD Huckins JN Jones-Lepp TL Getting DT Goddard JP Manahan SE 2004 Development of a passive, in situ, integrative sampler for hydrophillic organic contaminants in aquatic environments. Environ. Toxicol. Chem. 23, 1640–1648. (10.1897/03-603) 15230316

[25] Lohmann R Muir D 2010 Global aquatic passive sampling (AQUA-GAPS): using passive samplers to monitor POPs in the waters of the world1. Environ. Sci. Technol. 44, 860–864. (10.1021/es902379g) 20104908

[26] Matzke MM Allan SE Anderson KA Waters KM 2012 An approach for calculating a confidence interval from a single aquatic sample for monitoring hydrophobic organic contaminants. Environ. Toxicol. Chem. 31, 2888–2892. (10.1002/etc.2014) 22997050 PMC3581149

[27] Dropbox Inc. 2011 *Dropbox. In. 1.2.49 edn* San Francisco, CA: Dropbox. https://www.dropbox.com/release_notes.

[28] EPA. 2002 *National recommended water quality criteria: 2002*. US Environmental Protection Agency, Federal register document 02-32770. For more information see http://www.epa.gov/fedrgstr/EPA-WATER/2002/December/Day-27/w32770.htm.

[29] USEPA. 1992 Guidelines for exposure assessment. Washington, DC: U.S. Environmental Protection Agency.

[30] Hill IR 1989 Aquatic organisms and pyrethroids. Pest. Sci. 27, 429–457. (10.1002/ps.2780270408)

[31] Liu W Gan J Lee S Werner I 2005 Isomer selectivity in aquatic toxicity and biodegradation of bifenthrin and permethrin. Environ. Toxicol. Chem. 24, 1861–1866. (10.1897/04-457R.1) 16152954

[32] Weston DP Asbell AM Hecht SA Scholz NL Lydy MJ 2011 Pyrethroid insecticides in urban salmon streams of the Pacific Northwest. Environ. Pollut. 159, 3051–3056. (10.1016/j.envpol.2011.04.008) 21592636

[33] Weston D Holmes R You J Lydy M 2005 Aquatic toxicity due to residential use of pyrethroid insecticides. Environ. Sci. Technol. 39, 9778–9784. (10.1021/es0506354) 16475366

[34] Smith S Jr Lizotte RE Jr 2007 Influence of selected water quality characteristics on the toxicity of λ-Cyhalothrin and γ-Cyhalothrin to *Hyalella azteca*. Bull. Environ. Contam. Toxicol. 79, 548–551. (10.1007/s00128-007-9253-0) 17676253

[35] Foujut TL Rering C Tjeerdema RS 2011 Water quality criteria report for permethrin phase III: application of the pesticide water quality criteria methodology. Report prepared for the Central Valley Regional Water Quality Control Board. Davis, CA: University of California.

[36] Mamood AN Waller GD 1990 Recovery of learning responses by honeybees following a sublethal exposure to permethrin. Physiol. Entomol. 15, 55–60. (10.1111/j.1365-3032.1990.tb00492.x)

[37] Walker WJ Schreier CG Pucik LE 2004 Use of degradation rates of DDT and lindane isomers for determining the timing of release to sediments of Greens Bayou: Houston Ship Channel, Taxas. Environ. Forensics 5, 45–57. (10.1080/15275920490424033)

